# Artificial Intelligence–Based Video Assessment of Neonatal State

**DOI:** 10.1001/jamanetworkopen.2024.55948

**Published:** 2025-01-23

**Authors:** Monami Nishio, Naohisa Takeda, Ryutaro Miyata, Yushi Ito, Tetsuya Isayama, Shoi Shi, Yuka Wada

**Affiliations:** 1 National Center for Child Health and Development, Tokyo, Japan; 2 Fvital Inc, Tokyo, Japan; 3 University of Tsukuba, Tsukuba, Ibaraki, Japan

## Abstract

This case series study evaluates the accuracy of an automated video tool for monitoring neonatal states and tracking developmental changes.

## Introduction

The appropriate ratio of arousal state is a critical landmark of healthy development in neonates.^1^ However, manual assessment of arousal state is labor-intensive and impractical in clinical settings. Artificial intelligence (AI)–based video monitoring offers a potential solution, enabling more frequent and precise observation. Our study presents an automated video tool for monitoring neonatal states, evaluating its efficacy in tracking developmental changes.

## Methods

This case series study was approved by the National Center for Child Health and Development, with written informed consent obtained from parents. Detailed methods can be found in the eMethods of Supplement 1. Videos were captured using smartphones during the patient’s stay in the neonatal intensive care unit. Each patient was recorded 1 to 4 times during their stay. In total, 49 time point video records are included (eTable in Supplement 1). We used YOLOv7,^2^ an object detection model, and DWPose,^3^ a pose estimation model, for body part detection. Detection examples are shown in the eFigure in Supplement 1. We analyzed correlations between the speed and variance of head and hand movements captured in the videos and the neonatal arousal states^4^ assessed by nurses. We then tracked the developmental changes in movement speed and variance from postmenstrual weeks 34 to 44. Statistical analysis was performed from June 2023 to May 2024 using Python version 3.12.1 (Python Software Foundation). Two-sided *P* < .05 was considered statistically significant.

## Results

We included 27 Japanese patients (14 males; mean [SD] birth weight, 985 [488] g). Among the 5 patients with intracranial hemorrhage, 2 also had hydrocephalus. Both body part detection models demonstrated notable proficiency in detecting patients’ heads (head precision >0.95; recall >0.85) and were reasonably accurate for hands (hand precision >0.5; recall >0.85). As DWPose outperformed YOLOv7 in all evaluation metrics, we chose to proceed with DWPose for further analysis.

**Figure 1.**
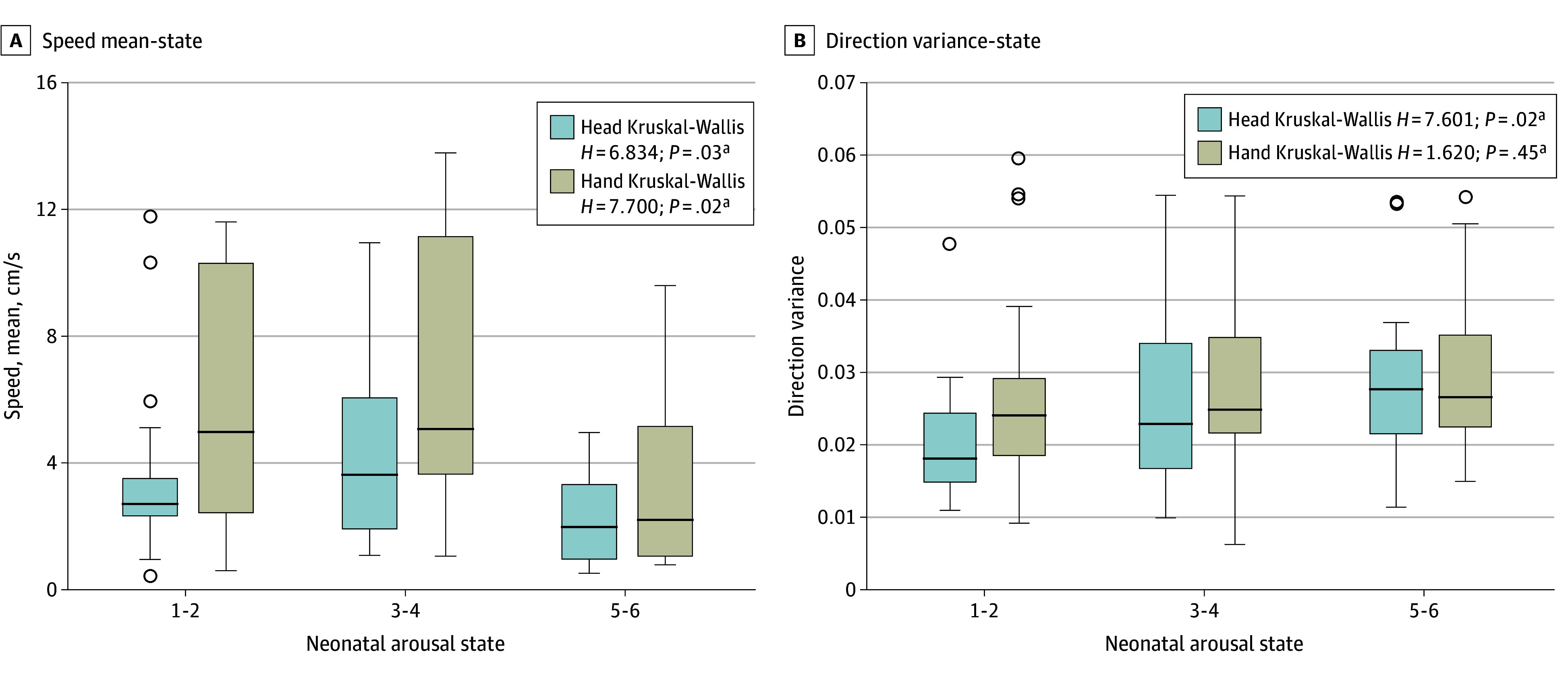
State Estimation Based on Video A, Correlation between neonatal arousal states, as defined by the Brazelton Neonatal Behavior Assessment Scale and labeled by nurses, and mean movement speeds of head and hand assessed from videos (head *H* = 6.834, *P* = .03; hand *H* = 7.700, *P* = .02, Kruskal-Wallis test). B, Correlation between neonatal arousal states and mean direction variances of head and hand assessed from videos (head *H* = 7.601, *P* = .02; hand *H* = 1.620, *P* = .445, Kruskal-Wallis test). Videos were recorded for 6 patients, totaling 1069 seconds.

First, we observed that the mean speed of the head and hands was significantly higher during neonatal arousal states 3 and 4 compared with other arousal states, suggesting that periods of wakefulness without crying may involve rapid movements (Figure 1A). In contrast, the variance in head movement direction showed a significant linear correlation with states, being particularly higher in states 5 and 6, implying that random and irregular movements are observed in higher states (Figure 1B).

**Figure 2.**
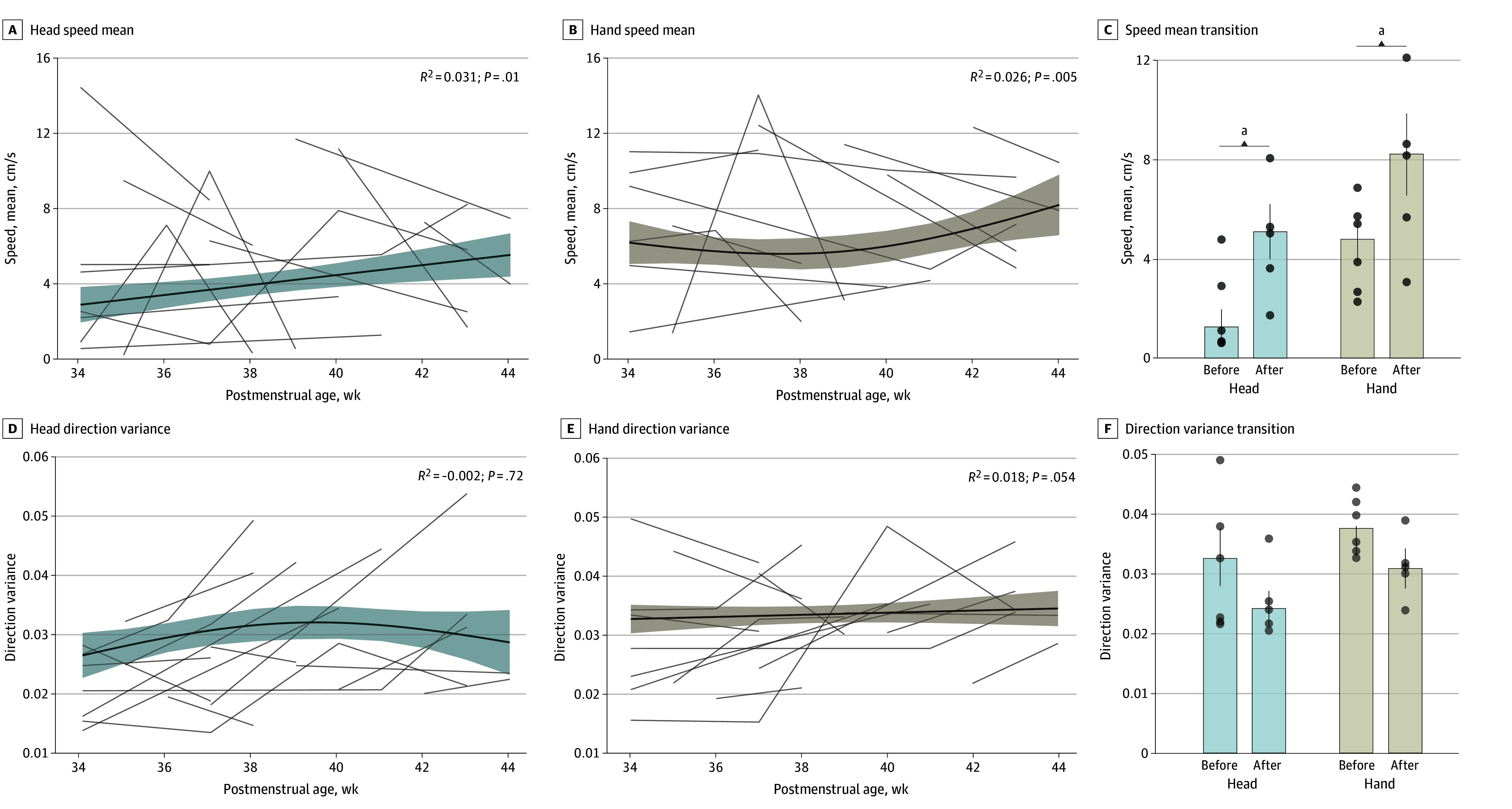
State Transition Through Development Transition of head (A) and hand (B) movement speeds across postmenstrual weeks 34 to 44. The mixed-effects model estimated the developmental trajectories of mean speed for the head (A) and hand (B), accompanied by a 95% CI shown as shaded areas (head: *R^2^* = 0.031, *P* = .01; hand: *R^2^* = 0.026, *P* = .005). The gray lines indicate the trajectories of each individual (N = 27). C, Comparison of mean speeds for each week before 40 weeks (weeks 34-39, 6 weeks) and after 40 weeks (weeks 40-44, 5 weeks) (head *t* = −2.478, *P* = .04; hand *t* = −2.180, *P* = .050, independent *t* test). Transition of (D) hand and head (E) direction variances across weeks 34 to 44. The mixed-effects model estimated the developmental trajectories of mean speed for the head (D) and hand (E), accompanied by a 95% CI shown as shaded areas (head: *R^2^* = −0.002, *P* = .72; hand: *R^2^* = 0.018, *P* = .054). The gray lines indicate the trajectories of each individual (N = 27). F, Comparison of direction variances for each week before 40 weeks (weeks 34-39, 6 weeks) and after 40 weeks (weeks 40-44, 5 weeks) (head *t* = 1.988, *P* = .08; hand *t* = 1.703, *P* = .12, independent *t* test). ^a^*P* < .05.

We observed a significant progressive increase in the speed of head and hand movements from weeks 34 to 44 (Figures 2A and 2B). The mean speed after a postmenstrual age of 40 weeks was significantly higher than before 40 weeks for both the head and hands (Figure 2C). Conversely, the variance in head and hand movements shows no statistical difference across developmental stages (Figures 2D and 2E) and remains consistent before and after a postmenstrual age of 40 weeks (Figure 2F).

## Discussion

In this study, we introduced a method to automatically track the speed and direction variances of neonatal body parts from videos. We found that mean speed, which is high in states 3 and 4, increases with development, whereas direction variance, which is high in states 5 and 6, does not show a significant difference across development. These findings align with reports that arousal state and state regulation improve with development,^1,5^ leading to more states 3 and 4 and fewer states 5 and 6.^5^ However, a limitation of this study is that the increase in movement may also reflect other factors such as the transition from preterm to writhing movements.^5^ Additionally, the small sample size calls for longitudinal studies with larger datasets to validate our findings. In summary, videos from standard smartphones can effectively assess neonatal arousal state, enabling efficient and practical monitoring of neonatal development in clinical settings.

## Notes

Supplement 1. **eMethods.** Supplementary Methods **eTable.** Time Points of Video Recording for Each Patient **eFigure.** A Sample of Deep Learning-Based Body Part Detection

Supplement 2.
Data Sharing Statement
