# Supplementary material for: Artificial Intelligence–Based Video Assessment of Neonatal State
Source: JAMA Netw Open. 2025 Jan 23;8(1):e2455948. doi: 10.1001/jamanetworkopen.2024.55948 (PMC11759000; doi:10.1001/jamanetworkopen.2024.55948)
Supplement: Supplement 1. — eMethods. Supplementary Methods eTable. Time Points of Video Recording for Each Patient eFigure. A Sample of Deep Learning-Based Body Part Detection [file jamanetwopen-e2455948-s001.pdf]

Supplemental Online Content

Nishio M, Takeda N, Miyata R, et al. Artificial intelligence–based video assessment of neonatal state. *JAMA Netw Open*. 2025;8(1):e2455948.
doi:10.1001/jamanetworkopen.2024.55948

eMethods. Supplementary Methods

eTable. Time Points of Video Recording for Each Patient

eFigure. A Sample of Deep Learning-Based Body Part Detection

This supplemental material has been provided by the authors to give readers additional information about their work.

© 2025 Nishio M et al. *JAMA Network Open*.

eMethods. Supplemental Methods

Video Collection

The smartphones (Moto G30, Motorola Trademark Holdings, LLC) were mounted on the frame of the infant warmer or cot, positioned to capture the patients' entire body. The videos were recorded at a resolution of 1920 pixels × 1080 pixels and at 30 frames per second.

Body Detection

Given that the head and hands are the most visible body parts in NICU settings, we developed a model to detect and track these specific areas.

Training:

For YOLO training, 200 frames from videos of four patients were annotated for the head, right hand, and left hand by a single annotator. For DWPose, we utilized a publicly available pre-trained model.

Validation:

To evaluate accuracy, we used 100 new frames from three patients who were not included in the training dataset. A single annotator manually assessed whether the regions of interest (ROIs) marked by the model overlapped with the actual locations of the body parts (head and hands). If the ROI was marked outside the actual location, it was counted as a false positive. If the model failed to detect a body part, it was counted as a false negative. Using this information, we calculated the accuracy, precision, and recall for each model.

Post-Processing

For each body part, we calculate the average x and y coordinates across the entire video segment for each time point, and we remove the frames where the x and y coordinate values exceed two standard deviations from the average. Additionally, if the x and y coordinates of the head or both hands are occluded, that frame is excluded from the analysis.

Movement Feature Calculation

Mean Speed:

Mean speed was calculated by measuring the distance the body part moved between consecutive frames (pixels/frame) and then summing this over 30 frames to express it in pixels/second. Pixels were converted to centimeters using the hand size of a patient (6 cm) as the scaling factor. Since depth information was not available, the centimeters are based on a 2D measurement.

Variance of Vector:

To calculate the variance, defined as the ratio of the magnitude of a resultant vector (formed by adding unit vectors) to the magnitude of a unit vector, we first summed the unit vectors over each second (30 frames), accounting for their angles. We then determined the magnitude of the resultant vector. The mean speed and variance of the vectors for the left and right hands were averaged per second. If only one of the two hands is occluded, the value from the other hand is used for the subsequent analysis.

Correlation with State

Labeling by Nurses:

We manually extracted 34 minutes and 7 seconds of video from six patients showing various states. Three NICU nurses annotated the neonatal state using the Neonatal Behavioral Assessment Scale (NBAS), which is a six-step scale widely employed in clinical settings to assess neonatal arousal states (see eTable 3). The nurses used a visual analog scale to event-code the segments corresponding to the same arousal state. We then broke down these event coding results to a per-second basis. Time segments were included only when at least two out of three nurses agreed on the state, resulting in 1,069 seconds (52.21%). The median value, which reflects the agreement of at least two nurses, was used to represent the arousal state for each second.

© 2025 Nishio M et al. *JAMA Network Open*.

Statistical Analysis:

We applied the body detection model to the videos and extracted the mean speed and variance for each second. The video segments were then classified based on their labeled states into three categories: 1 or 2, 3 or 4, and 5 or 6. We performed the Kruskal-Wallis test across the video clips (seconds) categorized into these three classes.

© 2025 Nishio M et al. *JAMA Network Open*.

eTable. Time points of video recording for each patient

The circles indicate the postmenstrual weeks when the videos were recorded for each patient.

Postmenstrual Age (Weeks)	Participants ID																										
	1	2	3	4	5	6	7	8	9	10	11	12	13	14	15	16	17	18	19	20	21	22	23	24	25	26	27
34				●	●		●	●				●									●	●			●		
35	●	●															●										
36							●				●																
37	●		●		●	●		●	●			●				●					●		●				
38		●	●				●			●																	
39	●					●											●	●								●	
40												●	●		●												
41				●																				●	●		●
42			●										●	●						●							
43				●								●			●	●			●								
44																							●				

© 2025 Nishio M et al. *JAMA Network Open*.

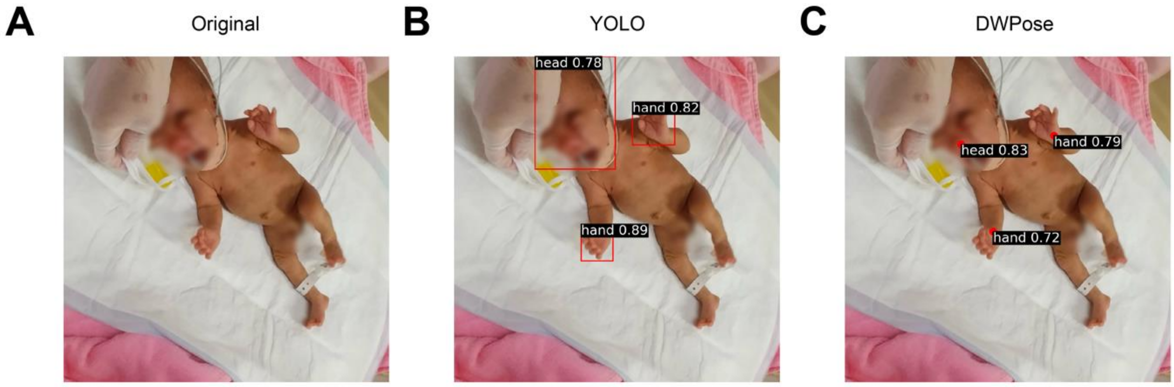

eFigure. A sample of deep learning-based body part detection

(A) The original sample image. (B) The image displaying the YOLO detection results with red bounding boxes. The name of each body part and the confidence of the detection are indicated for each body part within a black box. (C) The image displaying the DWPose detection results with red points. The name of each body part and the confidence of the detection are indicated for each body part within a black box.

© 2025 Nishio M et al. *JAMA Network Open*.
