# Supplementary material for: Artificial Intelligence–Based Video Assessment of Neonatal State
Source: JAMA Netw Open. 2025 Jan 23;8(1):e2455948. doi: 10.1001/jamanetworkopen.2024.55948 (PMC11759000; doi:10.1001/jamanetworkopen.2024.55948)
Supplement: Supplement 2. — Data Sharing Statement [file jamanetwopen-e2455948-s002.pdf]

Data Sharing Statement

Nishio. Artificial Intelligence–Based Video Assessment of Neonatal State. *JAMA Netw Open*. Published January 23, 2025. doi:10.1001/jamanetworkopen.2024.55948

Data
Data available: No

Additional Information
Explanation for why data not available: Our dataset includes videos of newborns, which are highly personal and cannot be shared.
